# Association between homocysteine and severe cerebral small vessel disease burden in patients with type 2 diabetes mellitus

**DOI:** 10.3389/fendo.2025.1640882

**Published:** 2026-01-06

**Authors:** Wensheng Huang, Huijuan Jie, Jinsheng Yi, Qingchang Liu, Simin Li, Yingying Luo, Shutong Tang, Zelin Chi, Changquan Wu

**Affiliations:** 1 Department of Radiology, The Seventh Affiliated Hospital of Sun Yat-sen University, Shenzhen, China; 2 Department of Rehabilitation Medicine, The Seventh Affiliated Hospital of Sun Yat-sen University, Shenzhen, China; 3 Department of Neuromedicine Center, The Seventh Affiliated Hospital of Sun Yat-sen University, Shenzhen, China; 4 Gastroenterology Department 2, The Seventh Affiliated Hospital of Sun Yat-sen University, Shenzhen, China

**Keywords:** body mass index, cerebral small vessel disease burden, homocysteine, microvascular, type 2 diabetes mellitus

## Abstract

**Objective:**

Cerebral small vessel disease (CSVD) is one of the common complications in patients with type 2 diabetes mellitus (T2DM). Homocysteine (Hcy), an emerging biomarker, has an unclear relationship with the CSVD burden in T2DM patients. This study aims to investigate the association between Hcy levels and the burden of severe CSVD in diabetic patients.

**Methods:**

A total of 236 patients with T2DM were enrolled in this study. Based on the total CSVD burden score, patients were divided into a mild CSVD burden group (score ≤ 2, n=181) and a severe CSVD burden group (score > 2, n=55). Multivariable logistic regression models were used to evaluate the association between Hcy levels and severe CSVD burden in T2DM patients. Restricted cubic spline (RCS) analyses were conducted to explore the nonlinear relationship between Hcy and the risk of severe CSVD burden. Subgroup analyses and interaction tests were performed to assess potential differences across groups.

**Results:**

Multivariable logistic regression analysis demonstrated that higher Hcy levels were independently associated with an increased risk of severe CSVD burden (OR = 1.13, 95% CI: 1.04–1.23). RCS analysis indicated a positive linear relationship between Hcy levels and the risk of severe CSVD burden. Subgroup analyses showed that this association remained significant in patients with BMI < 25 kg/m^2^, age ≥ 60 years, diabetes duration < 10 years, regardless of HbA1c levels, with or without hypertension, and in those without coronary artery disease or diabetic retinopathy. Moreover, a significant interaction was observed between BMI and the relationship between Hcy and severe CSVD burden (P for interaction = 0.020). The association was particularly pronounced in the BMI < 25 kg/m^2^ subgroup (OR = 1.26, 95% CI: 1.08–1.46).

**Conclusion:**

Elevated serum Hcy is independently associated with a higher burden of severe CSVD in patients with T2DM. Monitoring and managing Hcy levels may have potential value for identifying patients at greater risk of CSVD progression.

## Highlights

- The study found that higher serum Hcy levels were associated with an increased burden of cerebral small vessel disease (CSVD) in individuals with type 2 diabetes mellitus.
- Subgroup analysis revealed that the link between Hcy and severe CSVD was particularly pronounced in patients with a lower BMI.
- The findings suggest that monitoring Hcy levels could help identify T2DM patients at higher risk of CSVD, especially those with lower BMI.

## Introduction

Type 2 diabetes mellitus (T2DM) is highly prevalent among middle-aged and older adults and has become a major global public health concern, with its incidence continuing to rise worldwide (1, 2). In addition to well-recognized microvascular complications such as retinopathy and nephropathy, increasing evidence indicates that the cerebral microvasculature is also a major target of T2DM–related injury (3, 4). T2DM is associated with widespread cerebral microvascular dysfunction, including increased blood–brain barrier permeability, impaired cerebral blood-flow regulation, and structural alterations in white matter, which collectively contribute to imaging features of cerebral small vessel disease (CSVD) such as white matter hyperintensities (WMH), lacunes, and cerebral microbleeds (5). CSVD encompasses a spectrum of pathological processes affecting small cerebral arteries, arterioles, capillaries, and venules, ultimately leading to structural and functional microvascular abnormalities and a variety of clinical, radiological, and pathological manifestations (6, 7).

Previous studies have shown that individuals with T2DM have a higher prevalence of CSVD (8–10), and Mendelian randomization studies further support a causal relationship between T2DM and CSVD-related outcomes (11, 12). CSVD is a major contributor to stroke in patients with T2DM, accounting for nearly 30% of stroke-related mortality in this population (10). Given its high prevalence and substantial impact on functional outcomes, identifying modifiable factors associated with CSVD in T2DM is critical for early detection and prevention.

In recent years, elevated serum homocysteine (Hcy) has emerged as an independent risk factor for cardiovascular and cerebrovascular diseases, accelerating microvascular injury through oxidative stress and endothelial dysfunction (13, 14). Hcy is a sulfur-containing amino acid and a key intermediate in the methionine metabolism pathway. Biochemical evidence indicates that insulin promotes Hcy metabolism by activating cystathionine β-synthase (CBS), whereas the insulin resistance characteristic of T2DM disrupts this pathway, leading to reduced Hcy clearance (15, 16). Moreover, mild renal impairment and chronic low-grade inflammation—both commonly present in T2DM—may further increase circulating Hcy levels (17–19). Based on these diabetes-related metabolic disturbances, we hypothesized that Hcy functions not merely as a general vascular risk factor, but acts synergistically with the specific metabolic milieu of T2DM to accelerate the progression of severe CSVD burden.

Although previous studies have linked elevated Hcy to specific radiological features such as white matter hyperintensity burden, cerebral microbleeds, and lacunar infarctions (20, 21), these individual markers may not fully capture the cumulative severity of the disease. Compared with single imaging indicators, the total CSVD burden score provides a more comprehensive reflection of the global impact of microvascular damage on the brain (22); yet, evidence specifically linking Hcy to this composite burden within the T2DM population remains scarce. Therefore, the present study aimed to investigate the association between serum Hcy levels and the severity of total CSVD burden in patients with T2DM. We further evaluated potential nonlinear associations using restricted cubic spline (RCS) analyses and performed subgroup analyses to identify demographic or metabolic factors that may modify this association. These findings may provide new insights into the role of Hcy in CSVD pathophysiology among individuals with T2DM and support more precise cerebrovascular risk stratification in clinical practice.

## Methods

### Study design and population

This study retrospectively included patients with T2DM who visited our hospital between January 2022 and December 2024. The diagnosis of T2DM was based on criteria established by the World Health Organization (WHO) or the American Diabetes Association (ADA) (23, 24). Inclusion criteria were as follows: (1) confirmed diagnosis of type 2 diabetes; (2) age≥18 years; and (3) completion of cranial magnetic resonance imaging (MRI). Exclusion criteria included: (1) acute cerebrovascular events; (2) severe cardiac, hepatic, or renal failure; (3) malignancy; (4) known hereditary disorders affecting Hcy metabolism or current use of medications that significantly alter Hcy levels; and (5) pregnancy or lactation. This study was conducted in accordance with the principles of the Declaration of Helsinki and was approved by the Ethics Committee of the Seventh Affiliated Hospital of Sun Yat-sen University (Approval No. KY-2025-108-01), the requirement for informed consent was waived by the ethics committee.

### Data collection

Patient demographic characteristics, disease duration, comorbidities, and laboratory parameters were collected through the electronic medical record system. Collected data included age, sex, body mass index (BMI), duration of diabetes, presence of hypertension, coronary artery disease, and diabetic retinopathy. Laboratory assessments included Hcy, glycated hemoglobin (HbA1c), blood glucose, white blood cell count, lymphocyte count, neutrophil count, platelet count, erythrocyte sedimentation rate (ESR), serum uric acid, and urinary albumin.

### Brain MRI scanning and CSVD total burden assessment

**Figure 1:**
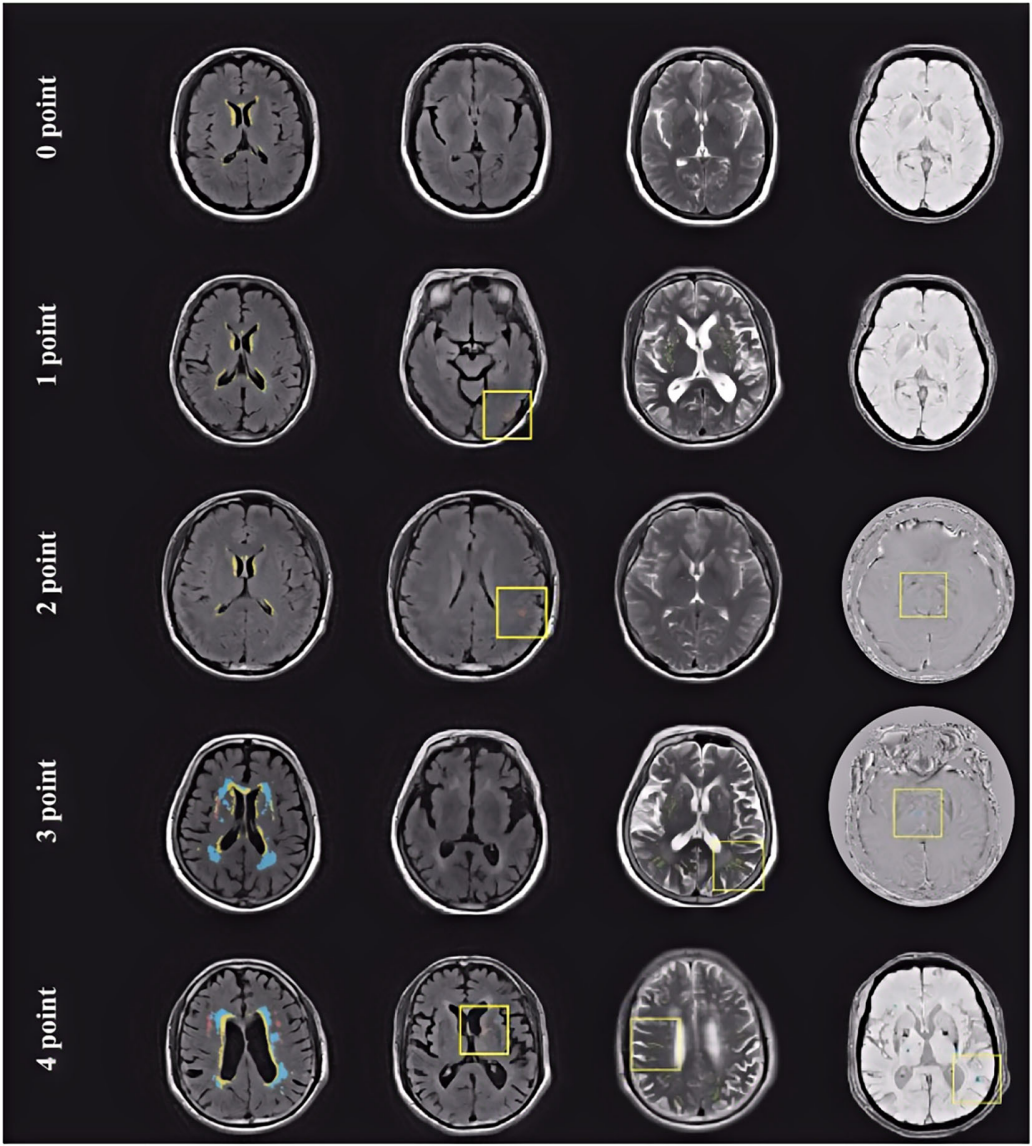
MRI images from patients with different cerebral small vessel disease scores.

All participants underwent cranial MRI examinations, with scanning sequences including at least T1-weighted imaging (T1WI), T2-weighted imaging (T2WI), fluid-attenuated inversion recovery (FLAIR), and susceptibility-weighted imaging (SWI) and/or diffusion-weighted imaging (DWI). MRI scans were performed using a 3.0T scanner (Siemens Skyra) to obtain T1WI, T2WI, FLAIR, and SWI sequences. CSVD imaging markers were independently assessed by two experienced neuroradiologists who were blinded to the participants’ clinical data. In cases of disagreement on any CSVD imaging marker, a third reviewer evaluated the images to reach a consensus. The total CSVD burden was evaluated using a composite scoring system incorporating four imaging biomarkers: white matter hyperintensities (WMH, graded using the Fazekas scale), lacunes, cerebral microbleeds (CMBs), and enlarged perivascular spaces (EPVS) in the basal ganglia (8, 25). Each marker was assigned a score based on its presence or severity, and the sum constituted the total CSVD burden score. The specific criteria were as follows: (1) White Matter Hyperintensities (WMH): 1 point for moderate-to-severe (Fazekas grade 2-3 for deep WMH, or Fazekas grade 3 for periventricular WMH); (2) Lacunes: 1 point for the presence of one or more lacunes; (3) Cerebral Microbleeds (CMBs): 1 point for the presence of one or more CMBs; and (4) Enlarged Perivascular Spaces (EPVS) in the basal ganglia: 1 point for moderate-to-severe (>10 in one hemisphere). The total point ranging from 0 to 4 (25) (**Figure 1**). Based on this total score, patients were divided into a mild CSVD burden group (score ≤ 2) and a severe CSVD burden group (score > 2).

### Statistical analysis

Data were analyzed using SPSS (version 26.0, IBM Corp.) and R software (version 4.2.2). Continuous variables were presented as mean (standard deviation) if normally distributed, and group comparisons were performed using the independent samples t-test. For non-normally distributed data, values were expressed as median (interquartile range) [M_50_ (P_25_, P_75_)], and the Mann-Whitney U test was used for between-group comparisons. Categorical variables were expressed as counts and percentages [n(%)], and comparisons were conducted using the chi-square test or Fisher’s exact test as appropriate. Multivariable logistic regression was used to analyze the association between Hcy levels and severe CSVD burden. Three regression models were constructed: Model 1 was unadjusted; Model 2 adjusted for age, sex, BMI, duration of diabetes, history of hypertension, history of coronary artery disease, and diabetic retinopathy; Model 3 further adjusted for serum uric acid, HbA1c, neutrophil count, platelet count, ESR, and urinary albumin on the basis of Model 2. The strength of association was expressed as odds ratios (ORs) with 95% confidence intervals (CIs). A two-sided P value < 0.05 was considered statistically significant.

Displayed are axial MRI images from patients with CSVD scores ranging from 0 to 4 points. Blue areas indicate white matter hyperintensities (WMH), and yellow boxes indicate other key imaging lesions contributing to the CSVD score in each case. 0 point: A 49-year-old female with no detectable abnormalities on MRI; 1 point: A 62-year-old female with a lacunar infarct in the left occipital lobe; no other abnormalities observed; 2 points: A 45-year-old male with a lacunar infarct in the left parietal lobe and a CMB in the left frontal lobe; 3 points: A 68-year-old female with periventricular WMH rated Fazekas grade 2, EPVS in the left parietal lobe, and a CMB in the left basal ganglia; 4 points: A 66-year-old male with periventricular WMH (Fazekas grade 2), a lacunar infarct in the left basal ganglia, EPVS in the right frontal lobe, and a CMB in the left temporal lobe.

## Results

### Characteristics of the study population

A total of 236 patients with T2DM were included in this study, with a mean age of 60.80 ± 9.93 years; 159 (67.37%) were male. Based on the total CSVD burden score, 181 patients (76.70%) were classified into the mild CSVD burden group, and 55 patients (23.30%) into the severe CSVD burden group. Compared with the mild CSVD burden group, patients in the severe group were significantly older (P < 0.001). The prevalence of hypertension and coronary artery disease was significantly higher in the severe group, which also showed higher levels of Hcy and lower levels of HbA1c (all P < 0.05). No significant differences were observed between the two groups in other characteristics (all P > 0.05). Detailed results are presented in **Table 1**.

**Table 1:** Characteristics of participants between mild CSVD burden and severe CSVD burden.

Variables	Total (n = 236)	Mild CSVD burden group (n = 181)	Severe CSVD burden group (n = 55)	Statistic	*P*
Age, years	60.80 ± 9.93	59.31 ± 9.72	65.69 ± 9.05	t=-4.33	<0.001
Gender, n(%)				χ^2^=1.68	0.195
Male	159 (67.37)	118 (65.19)	41 (74.55)		
Female	77 (32.63)	63 (34.81)	14 (25.45)		
BMI, kg/m^2^	25.08 ± 3.28	25.19 ± 3.44	24.71 ± 2.68	t=0.94	0.349
Duration of diabetes, years				χ^2^=0.22	0.636
< 10	131 (55.51)	102 (56.35)	29 (52.73)		
≥ 10	105 (44.49)	79 (43.65)	26 (47.27)		
Hypertension, n(%)				χ^2^=8.29	0.004
No	81 (34.32)	71 (39.23)	10 (18.18)		
Yes	155 (65.68)	110 (60.77)	45 (81.82)		
Coronary artery disease, n(%)				χ^2^=5.34	0.021
No	155 (65.68)	126 (69.61)	29 (52.73)		
Yes	81 (34.32)	55 (30.39)	26 (47.27)		
Diabetic Retinopathy, n(%)				χ^2^=0.01	0.930
No	149 (63.14)	114 (62.98)	35 (63.64)		
Yes	87 (36.86)	67 (37.02)	20 (36.36)		
SBP, mmHg	131.22 ± 17.03	131.63 ± 16.87	129.87 ± 17.65	t=0.67	0.504
DBP, mmHg	80.08 ± 10.48	80.64 ± 10.10	78.27 ± 11.56	t=1.47	0.144
Insulin, μU/mL	8.18 (6.39, 12.48)	8.29 (6.42, 12.32)	7.69 (6.28, 12.95)	Z=-0.48	0.633
HbA1c, %	7.23 (6.50, 8.70)	7.40 (6.60, 9.00)	6.90 (6.30, 8.10)	Z=-2.45	0.014
Blood glucose, mmol/L	7.28 ± 2.54	7.28 ± 2.55	7.30 ± 2.52	t=-0.07	0.947
WBC, 10^9^/L	6.83 ± 2.14	6.70 ± 1.78	7.23 ± 3.02	t=-1.61	0.108
Lymphocytes, 10^9^/L	2.02 ± 0.78	2.02 ± 0.68	2.01 ± 1.06	t=0.12	0.908
Neutrophils count,10^9^/L	3.83 (2.93, 4.72)	3.75 (2.92, 4.72)	4.11 (3.29, 4.71)	Z=-1.25	0.212
Platelets count, 10^9^/L	214.81 ± 61.13	215.85 ± 61.27	211.38 ± 61.11	t=0.47	0.636
Hcy, μmol/L	11.85 ± 4.56	11.25 ± 4.03	13.84 ± 5.58	t=-3.79	<0.001
ESR, mm/h	7.00 (5.00, 8.00)	7.00 (5.00, 8.00)	7.00 (5.00, 8.00)	Z=-0.42	0.671
Serum uric acid, μmol/L	361.88 ± 95.72	361.32 ± 90.06	363.72 ± 113.28	t=-0.16	0.871
Urinary albumin, mg/L	7.25 (2.35, 17.53)	7.66 (2.40, 19.01)	4.58 (2.26, 16.90)	Z=-1.27	0.203

BMI, Body Mass Index; CSVD, Cerebral Small Vessel Disease; SBP, Systolic Blood Pressure; DBP, Diastolic Blood Pressure; HbA1c, Glycated Hemoglobin; WBC, White Blood Cell count; Hcy, Homocysteine; ESR, Erythrocyte Sedimentation Rate.

### The relationship between Hcy and CSVD burden

**Table 2** presents the results of multivariable regression analyses examining the association between Hcy and CSVD burden. A significant positive correlation between Hcy levels and the risk of severe CSVD was observed across all three models. In Model 3, each 1 μmol/L increase in Hcy was associated with a 13% higher risk of severe CSVD (OR = 1.13, 95% CI: 1.04-1.23). When analyzed by Hcy quartiles, the risk of severe CSVD was significantly higher in the highest quartile (Q4) compared to the lowest (Q1), with an OR of 6.23 (95% CI: 1.87–20.78). These findings indicate a robust and stable positive association between serum Hcy levels and the risk of severe CSVD burden in patients with T2DM.

**Table 2:** Association between Hcy and CSVD burden.

Variables	Model1		Model2		Model3	
	OR (95%CI)	*P*	OR (95%CI)	*P*	OR (95%CI)	*P*
Hcy	1.12 (1.05, 1.20)	0.001	1.09 (1.02, 1.18)	0.016	1.13 (1.04, 1.23)	0.005
Hcy quantile (µmol/L)						
Q1 (< 8.90)	Reference		Reference		Reference	
Q2 (8.90 - 11.17)	2.82 (0.92, 8.59)	0.069	2.60 (0.80, 8.48)	0.113	2.19 (0.65, 7.43)	0.180
Q3 (11.17 - 13.66)	3.93 (1.33, 11.57)	0.013	3.62 (1.12, 11.75)	0.032	3.30 (1.01, 11.10)	0.048
Q4 (> 13.66)	6.42 (2.23, 18.49)	<0.001	4.83 (1.52, 15.32)	0.007	6.23 (1.87, 20.78)	0.003

Model 1: Crude (unadjusted) model.

Model 2: Adjusted for sex, age, BMI, duration of diabetes, hypertension, coronary artery disease, and diabetic retinopathy.

Model 3: Adjusted for sex, age, BMI, duration of diabetes, hypertension, coronary artery disease, diabetic retinopathy, serum uric acid, HbA1c, neutrophil count, platelet count, ESR, and urinary albumin.

**Figure 2:**
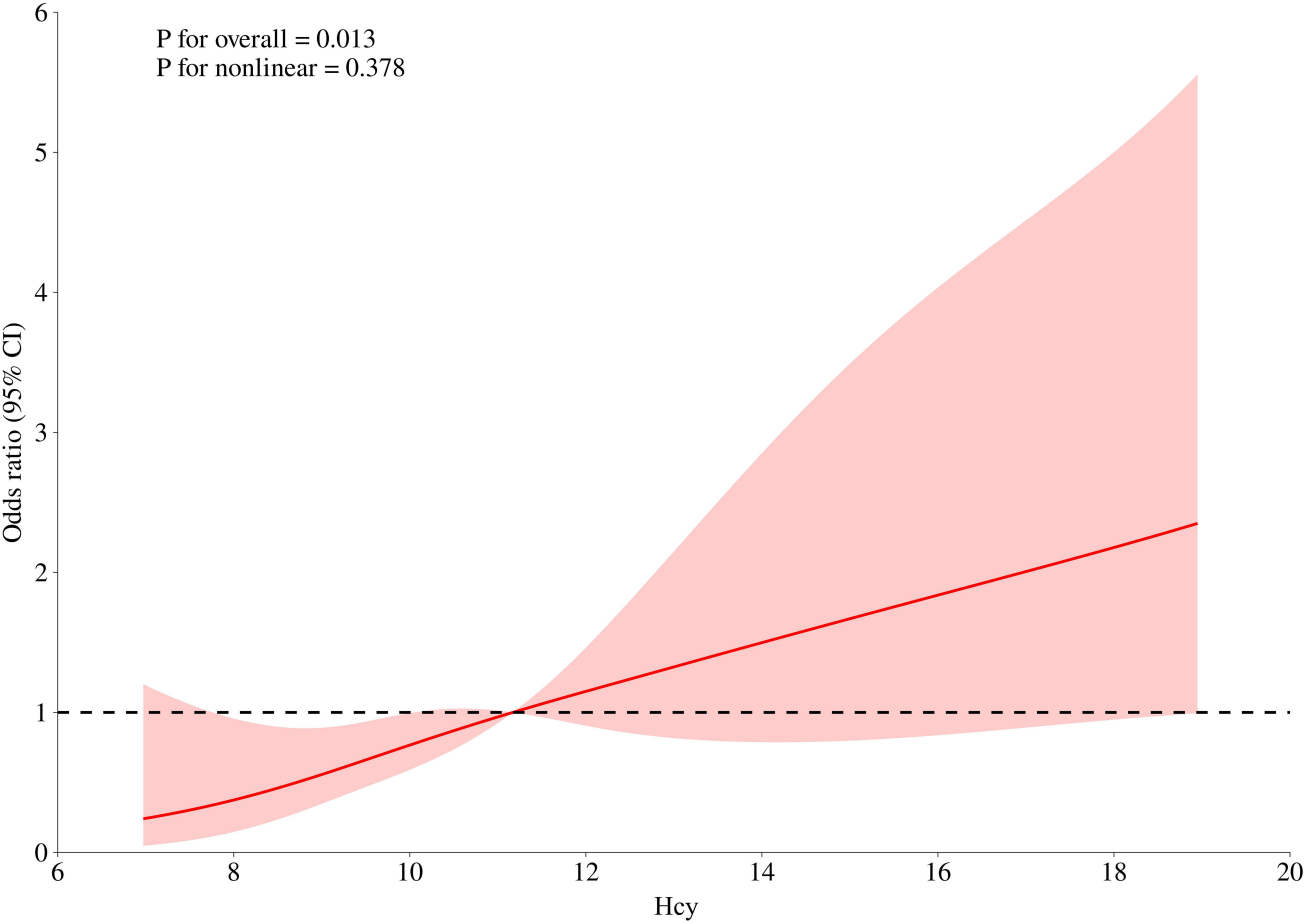
RCS plot of Hcy levels and CSVD burden. The red line represents the adjusted OR estimated from the RCS model (adjusted for covariates included in Model 3), and the shaded area represents the 95% confidence interval. Hcy, homocysteine; OR, odds ratio; CI, confidence interval; RCS, restricted cubic spline.

RCS analysis (**Figure 2**) demonstrated a positive, approximately linear relationship between serum Hcy levels and the risk of severe CSVD burden in patients with T2DM after adjusting for all covariates in Model 3 (P for overall = 0.013; P for non-linear = 0.378). This further indicates that the risk of severe CSVD burden continuously increases with rising Hcy levels.

### Subgroup analysis

To explore the potential effect modification of clinical characteristics on the association between Hcy levels and severe CSVD burden, subgroup analyses and interaction tests were conducted. A significant positive association between Hcy and severe CSVD burden was observed across multiple subgroups, including males, patients aged ≥60 years, those with BMI < 25 kg/m^2^, diabetes duration <10 years, individuals with hypertension, and those without coronary artery disease or diabetic retinopathy. This association remained significant regardless of glycemic control (HbA1c < 7% or ≥7%) (all P < 0.05). Interaction analysis indicated that BMI significantly modified the Hcy–CSVD relationship (P for interaction = 0.020). Results are summarized in **Table 3**.

**Table 3:** Subgroup analysis of the association between Hcy and CSVD burden.

Variables	n (%)	OR (95%CI)	P	P for interaction
Gender, n(%)				0.488
Male	159 (67.37)	1.16 (1.06 - 1.27)	0.001	
Female	77 (32.63)	1.06 (0.86 - 1.30)	0.604	
Age, years				0.710
< 60	109 (46.19)	1.13 (0.95 - 1.35)	0.165	
≥ 60	127 (53.81)	1.11 (1.02 - 1.21)	0.013	
BMI, kg/m^2^				0.020
< 25	121 (51.27)	1.26 (1.08 - 1.46)	0.003	
≥ 25	115 (48.73)	1.09 (0.99 - 1.19)	0.078	
Duration of diabetes, years				0.475
< 10	131 (55.51)	1.17 (1.03 - 1.33)	0.014	
≥ 10	105 (44.49)	1.11 (1.00 - 1.24)	0.054	
HbA1c, %				0.100
< 7	56 (23.73)	1.35 (1.07 - 1.70)	0.011	
≥ 7	180 (76.27)	1.10 (1.01 - 1.19)	0.032	
Hypertension, n(%)				0.763
No	81 (34.32)	1.14 (0.99 - 1.31)	0.072	
Yes	155 (65.68)	1.11 (1.01 - 1.21)	0.033	
Coronary artery disease, n(%)				0.586
No	155 (65.68)	1.16 (1.05 - 1.28)	0.003	
Yes	81 (34.32)	1.07 (0.94 - 1.22)	0.289	
Retinopathy, n(%)				0.411
No	149 (63.14)	1.14 (1.03 - 1.27)	0.011	
Yes	87 (36.86)	1.05 (0.90 - 1.22)	0.529	

## Discussion

We found that elevated serum Hcy levels were independently associated with an increased risk of severe total CSVD burden in patients with T2DM, with subgroup analyses indicating that this positive association was particularly prominent in those with lower BMI. These findings offer new insights into the pathophysiological mechanisms of CSVD in the diabetic population.

Our results are generally consistent with previous studies reporting associations between Hcy and cerebrovascular diseases. Hyperhomocysteinemia has been identified as an independent risk factor for atherosclerosis, stroke, and cognitive impairment (26–30). Other studies have also shown correlations between Hcy levels and both the volume and progression of white matter hyperintensities, as well as the occurrence of lacunar infarctions (31, 32).

Elevated Hcy levels are commonly observed in patients with T2DM, which may be related to factors such as insulin resistance and impaired renal function (33–35). Studies have suggested that in individuals with T2DM, Hcy may exacerbate the risk of both microvascular and macrovascular complications (36, 37). Moreover, Hcy has been associated with cognitive decline in T2DM patients, with CSVD potentially playing a mediating role in this relationship (38). A nomogram developed to predict mild vascular cognitive impairment in patients with T2DM also included Hcy levels and total CSVD burden score as key predictors in the model (39). By utilizing the total CSVD burden as a composite indicator, the present study systematically analyzed the diabetic population and found that Hcy levels were independently and dose-dependently associated with CSVD severity. These findings provide preliminary evidence supporting a potential role of Hcy in the pathological processes underlying CSVD in T2DM.

Elevated Hcy levels may exacerbate T2DM-specific vascular pathology through synergistic mechanisms, promoting severe CSVD distinct from traditional atherogenic pathways. First, in the hyperglycemic environment of T2DM, elevated Hcy levels coexist with advanced glycation end-products (AGEs). These metabolic toxicities exert synergistic deleterious effects on the vasculature. While hyperglycemia drives AGE-RAGE signaling, Hcy induces protein homocysteinylation and auto-oxidation. Together, these pathways amplify oxidative stress and inflammation, accelerating vascular endothelial injury beyond the damage caused by hyperglycemia alone (40). Second, Hcy impairs endothelial function by inhibiting nitric oxide (NO) bioavailability and increasing asymmetric dimethylarginine (41, 42). This direct endothelial toxicity and oxidative stress leads to blood-brain barrier disruption and matrix metalloproteinase imbalance, which directly contribute to the structural changes of CSVD (e.g., lacunes and WMH) (43, 44). Crucially, these mechanisms—direct endothelial dysfunction, BBB disruption, and AGEs-mediated damage—represent pathological pathways that are distinct from lipid-driven atherosclerosis (40, 41). This biological distinction suggests that Hcy exerts direct vascular toxicity through pathways that do not strictly depend on the presence of traditional dyslipidemia.

Our subgroup analysis revealed that the positive association between Hcy levels and severe CSVD burden in T2DM patients remained significant among males, those aged ≥ 60 years, individuals with BMI < 25 kg/m^2^, a diabetes duration < 10 years, regardless of HbA1c levels, those with hypertension, and those without coronary artery disease or diabetic retinopathy. Male patients may be more susceptible due to the lack of estrogen-mediated vascular protection and relatively higher Hcy levels, which may make the direct neurovascular toxicity of Hcy more pronounced, thereby strengthening its association with severe CSVD burden (45). In older T2DM patients, compromised baseline cerebrovascular integrity and higher Hcy levels may make it more likely for the deleterious effects of Hcy to surpass vascular compensatory mechanisms, leading to more severe CSVD. Crucially, we found that Hcy remained a significant risk factor for severe CSVD in both patients with well-controlled blood glucose (HbA1c < 7%) and those with poor control (HbA1c ≥ 7%). This suggests that Hcy contributes to microvascular damage through pathways distinct from hyperglycemia-induced injury, implying that Hcy remains a potent driver of CSVD progression even when glycemic targets are met. This independent pathogenic role aligns with our observations regarding disease chronicity. Among patients with a shorter duration of diabetes, Hcy may act as an earlier and more independent risk factor driving rapid CSVD progression, resulting in a more significant association. In contrast, among those with long-standing diabetes, multiple concurrent factors may collectively drive CSVD severity, diluting the specific contribution of Hcy. Hypertension can damage the endothelium through mechanical shear stress, while Hcy accelerates microvascular injury through oxidative stress and endothelial dysfunction. In H-type hypertension (i.e., hypertension with elevated Hcy), the total CSVD burden is markedly higher, suggesting a synergistic effect, thereby reinforcing the association between Hcy and CSVD in the hypertensive subgroup (46). Atherosclerotic macrovascular diseases such as coronary artery disease may introduce additional inflammatory and thrombotic mechanisms that obscure Hcy’s specific impact on the microvascular endothelium. Conversely, in patients without coronary artery disease, Hcy’s damage to small arteries and capillaries may be more clearly reflected in the CSVD burden (47). A cohort study stratified by diabetic retinopathy status reported a CSVD detection rate of 40.2% in patients with retinopathy, compared to only 30.1% in those without, indicating a greater systemic microvascular burden in those with retinopathy (48). In patients without retinopathy, the “competitive” injury from other microvascular complications is reduced, allowing the effects of Hcy such as increasing blood-brain barrier permeability and upregulating endothelial adhesion molecule expression-to more directly contribute to a higher CSVD burden (49).

An interesting finding of this study is that among patients with BMI < 25 kg/m^2^, each 1 μmol/L increase in Hcy was associated with a 26% higher risk of severe CSVD, whereas this association disappeared in obese individuals. Although the underlying mechanisms remain unclear, several hypotheses may help explain this observation. First, obesity itself is a well-established strong risk factor for CSVD (50, 51). In obese T2DM patients, an exaggerated pro-inflammatory and pro-oxidative state—combined with severe insulin resistance and adipokine dysregulation—may be the primary pathological drivers of CSVD (52–54). These dominant factors may mask or dilute the independent effect of Hcy in individuals with higher BMI. In contrast, in T2DM patients with normal weight, the overall burden of such confounders may be relatively lower, allowing Hcy to emerge as a more distinct and recognizable risk marker for CSVD. Second, adipose tissue also participates in Hcy metabolism, and differences in BMI may influence Hcy distribution and clearance (41, 52, 55, 56). This finding suggests that monitoring and targeting Hcy levels may hold greater clinical relevance for non-obese T2DM patients. However, larger-scale studies are needed to validate this association and further elucidate the underlying mechanisms.

The strength of our study lies in its focused evaluation of the association between serum Hcy levels and CSVD burden specifically in the T2DM population, addressing a gap in the current literature. Moreover, the subgroup analysis revealed a significant modifying effect of BMI on the Hcy-CSVD relationship, with a more pronounced effect of Hcy observed in non-obese individuals (BMI<25 kg/m^2^). As a convenient and cost-effective biomarker, serum Hcy may aid in identifying T2DM patients at elevated risk of CSVD, providing a basis for early warning and risk stratification. Hcy-lowering interventions targeting specific subpopulations of T2DM patients particularly those with lower BMI may represent a potential strategy for preventing or delaying CSVD progression.

However, this study has several limitations. First, as a cross-sectional analysis, it cannot establish a causal relationship. Second, the absence of a non-diabetic control group is a major limitation. This makes it difficult to determine whether the observed relationship between Hcy and CSVD is diabetes-specific or reflects broader vascular mechanisms present in non-diabetic individuals. Therefore, our findings are specific to the T2DM population. Third, the sample size was moderate and drawn from a single center, necessitating validation in large scale, prospective cohorts. Fourth, CSVD assessment relied solely on conventional MRI sequences without incorporating functional indicators such as cerebral perfusion metrics. Finally, some potential risk factors, such as detailed lipid profiles and specific renal function markers, were not measured, raising the possibility of residual confounding. Future longitudinal cohort and interventional studies are needed to verify these associations and evaluate the impact of Hcy-lowering strategies on the progression of CSVD in diabetic patients.

## Conclusion

This study is the first to demonstrate in a T2DM population that elevated Hcy levels are independently and linearly associated with an increased total CSVD burden. This association was particularly pronounced in patients with lower BMI. These findings suggest that Hcy levels may, to some extent, reflect the severity of CSVD in T2DM patients and hold potential as a biomarker for targeting this pathology. Clinically, attention should be given to monitoring Hcy levels in diabetic patients, incorporating it into cerebral microvascular risk assessment. Future prospective studies are warranted to further elucidate the mechanistic role of Hcy in CSVD development and progression, and to assess whether lowering Hcy levels can alleviate CSVD burden and improve cognitive outcomes.

## Data Availability

The original contributions presented in the study are included in the article/supplementary material. Further inquiries can be directed to the corresponding authors.
